# Genotype–Phenotype Correlations in 2q37-Deletion Syndrome: An Update of the Clinical Spectrum and Literature Review

**DOI:** 10.3390/genes14020465

**Published:** 2023-02-11

**Authors:** Eva-Cristiana Gavril, Irina Nucă, Monica-Cristina Pânzaru, Anca Viorica Ivanov, Cosmin-Teodor Mihai, Lucian-Mihai Antoci, Cristian-Gabriel Ciobanu, Cristina Rusu, Roxana Popescu

**Affiliations:** 1 Medical Genetics Department, Faculty of Medicine, “Grigore T. Popa” University of Medicine and Pharmacy, University Street, No 16, 700115 Iasi, Romania; 2 Investigatii Medicale Praxis, St. Moara de Vant No 35, 700376 Iasi, Romania; 3 Medical Genetics Department, “Saint Mary” Emergency Children’s Hospital, St. Vasile Lupu No 62, 700309 Iasi, Romania; 4 Pediatrics Department, Grigore T. Popa University of Medicine and Pharmacy, University Street No. 16, 700115 Iasi, Romania

**Keywords:** 2q37 deletion, genotype–phenotype correlations, brachydactyly E, intellectual disability

## Abstract

2q37 microdeletion/deletion syndrome (2q37DS) is one of the most common subtelomeric deletion disorders, caused by a 2q37 deletion of variable size. The syndrome is characterized by a broad and diverse spectrum of clinical findings: characteristic facial dysmorphism, developmental delay/intellectual disability (ID), brachydactyly type E, short stature, obesity, hypotonia in infancy, and abnormal behavior with autism spectrum disorder. Although numerous cases have been described so far, the exact mapping of the genotype and phenotype have not yet been achieved. Materials and Methods: In this study we analyzed nine newly diagnosed cases with 2q37 deletion (3 male/6 female, aged between 2 and 30 years old), and followed up at the Iasi Regional Medical Genetics Centre. All patients were tested first with MLPA using combined kits P036/P070 subtelomeric screening mix and follow-up mix P264; after, the deletion size and location were confirmed via CGH-array. We compared our findings with the data of other cases reported in the literature. Results: From nine cases, four had pure 2q37 deletions of variable sizes, and five presented deletion/duplication rearrangements (with chromosomes 2q, 9q, and 11p). In most cases, characteristic phenotypic aspects were observed: 9/9 facial dysmorphism, 8/9 global developmental delay and ID, 6/9 hypotonia, 5/9 behavior disorders, and 8/9 skeletal anomalies—especially brachydactyly type E. Two cases had obesity, one case had craniosynostosis, and four had heart defects. Other features found in our cases included translucent skin and telangiectasias (6/9), and a hump of fat on the upper thorax (5/9). Conclusions: Our study enriches the literature data by describing new clinical features associated with 2q37 deletion, and possible genotype–phenotype correlations.

## 1. Introduction

2q37 microdeletion/deletion syndrome (2q37DS), also known as Albright hereditary osteodystrophy-like syndrome or brachydactyly-intellectual disability syndrome, is a rare genetic disorder caused by a deletion of variable size in the long (q) arm of chromosome 2, in region 2q37. The syndrome is characterized by a broad and diverse spectrum of clinical findings. The most common phenotypic features include mild to moderate developmental delay/intellectual disability (ID), brachymetaphalangy of digits 3–5 (brachydactyly type E) [1,2,3], short stature, obesity, hypotonia in infancy, abnormal behavior with autism spectrum disorder (24–35%) [3,4,5], joint hypermobility, and scoliosis [6]. Most individuals with 2q37DS have a typical dysmorphic face: broad or rounded facies (41%), frontal bossing (35%), midface hypoplasia, thin, arched eyebrows with deep-set eyes, upslanting palpebral fissures, hypoplastic alae nasi, prominent columella, thin vermilion border, and minor ear defects. In 20–30% of cases, visceral malformations were also present: congenital heart disease (mostly septal defects), gastrointestinal or genitourinary anomalies, central nervous system malformations, renal anomalies, and Wilms tumors. Rarely, patients may have associated seizures, eczema, osteopenia, and hyperactivity with attention deficit disorder [2,6,7,8].

The 2q37 locus, one of the most commonly deleted subtelomeric regions, is divided in three sub-bands: 2q37.1, 2q37.2, and 2q37.3. The deletion of the terminal region of chromosome 2 can involve one or all three sub-bands, the deletion size being highly variable as well as the deletion type: interstitial, proximal, or distal, and terminal. Most cases have a terminal deletion ranging from 2 to 9 Mb [1,9]. 

In some individuals, 2q37 microdeletion is the result of chromosome rearrangements involving 2q37 (reciprocal translocations, inversions, ring chromosomes). To date, there is a limited number of cases with pure 2q37 deletion reported in the literature (~115 cases) [6,8].

At least 197 genes are located in the 2q37 region (230.7–243.2 Mb; Hg19; NCBI map viewer http://www.ncbi.nlm.nih.gov/mapview/ accessed on 10 October 2022); for the majority of them, implication in 2q37DS is still unknown, and only a few of them have been correlated with the potential 2q37-deletion phenotype [5,6,10,11,12,13]. *HDAC4* -is considered responsible for many of the phenotypical findings in 2q37DS, including brachymetaphalangy and intellectual disability, behavioral disorders, and seizures [7]. While the role of *HDAC4* is quite well established, for other genes the involvement is merely suggested by the overlapping deleted region in reported cases: *GPC1*, *PDC1*, *STK25,* and *GPR35* are candidate genes for facial dysmorphism and brachydactyly, *FARP2*- for facial dysmorphism, brachydactyly and behavioral disorders, *TWIST2*- candidate genes for brachydactyly, behavioral disorders, various skeletal defects, and cardiac anomalies, *CAPN10*, *HDLBP*, *SH3BP4*, *AGAP1*-overweightness or obesity, *D2HGDH*-possible responsible seizures, and *KIF1A*-behavioral disorders [6,7,10,11,12,13].

Although more than 100 cases have been described worldwide, an exact mapping for genotype–phenotype correlations have not yet been possible because CGH-array, the golden standard for microdeletions, was used to establish the deletion size only in recent studies. In the older studies, the deletion was delineated only with chromosomal analysis or the FISH test. The low incidence of 2q37DS is accompanied by clinical and molecular variability, determining a rather limited accumulation of data in the specialty literature, and complicating the establishment of a genotype–phenotype correlation. Chromosomal anomalies are difficult to establish due to numerous factors such as deletion size, genes involved, complex chromosomal rearrangements, mosaicism, and genetic or non-genetic modifiers [14,15,16,17].

In this context, our paper aims to analyze the phenotypes of pure (homogeneous and mosaic) and complex 2q37 DS cases, in order to delineate the hallmark features of the syndrome and highlight new suggestive elements. We also aim to analyze the effect of associated CNV on phenotypes for a better selection of cases in which array CGH should be used.

## 2. Materials and Methods

In this study we analyzed 9 new cases with 2q37 deletion that were not reported until now in the literature, and we compared our results with the data previously recorded by other authors. 

We used MLPA screening kits to assess all patients at the Iasi Regional Medical Genetic Center with intellectual disability (mild to severe), developmental delay, distinctive facial dysmorphia, and/or multiple congenital defects. Patients with a 2q37 deletion met the study’s inclusion criteria. All 9 cases with 2q37 deletion that we identified were re-assessed, and then followed up with periodically. The ages of the patients included in study were between 2 and 30 years; 6 were females and 3 were males. In only 2 cases were the patients related (siblings), while in the other 7 cases, no relationship was established.

Before enrolment in the study, each individual provided their informed consent for inclusion. The protocol was accepted by the Ethical Committee of the University of Medicine and Pharmacy “Grigore T. Popa” Iasi (approved at 7 July 2019, No. 14629), and all the requirements of the Declaration of Helsinki were followed. Anonymization of patients’ personal data was carried out by assigning code numbers to each patient, and no personal information was disclosed unless it was necessary for the study. Before the start of the study, patients (or their legal guardians) gave their informed consent. Every patient voluntarily participated in the study.

For the clinical assessment of our patients, echocardiography, MRI, abdominal and pelvic ultrasound were used in all cases to establish the phenotypic defects. For the investigation of skeletal anomalies, Rx of the limbs were taken. The degree of intellectual disability and developmental delay was established by the Bayles Scale for Infant Development IV (0–42 months), DF-MOT (Motor developmental scales, 0–4 years), Neuropsychomotor Function Evaluation Battery-NP-MOT (4–12 years), and the Stanford–Binet and Wechsler Intelligence Scale V (children—6 years and adults); for autism spectrum disorders, the Autism Spectrum Rating Scales (ASRS) were used.

DNA extraction from peripheral blood was performed using the Wizard Genomic DNA Purification Kit (Promega Corp., Madison, WI, USA).

In this study, we initially detected 237 deletions using the MLPA (Multiplex ligation-dependent probe amplification) approach, utilizing P036/P070 subtelomeric screening probe mixes; subsequently, the 2q changes were confirmed by the follow-up mix P264 (MRC Holland, Amsterdam, The Netherlands), which contained 13 probes in the terminal 5.0 Mb of 2q37.3. 

MLPA analysis was performed, as previously described [14]. Briefly, 100 ng of genomic DNA was denatured and hybridized with selected subtelomeric probe mixes at 60 °C for 20 hours. After a ligation step at 54 °C for 15 min and a PCR protocol using Cy5-labeled universal primers contained in the kit, the fluorescent amplification products were separated by capillary electrophoresis in a CEQ 8000 GeXP Genetic Analysis System sequencer (Beckman Coulter, Brea, CA. USA). The DNA copy number was estimated using the Coffalyser.net program. Heterozygous deletions/duplications were established when recognition sequences had a 35–50% reduced/increased relative peak area of the amplification product of that probe.

Furthermore, CGH-array was performed in all cases to describe the deletion size better, and to identify other associated CNVs. 

CGH-array analysis was performed using the SurePrint G3 Human ISCA CGH+SNP Microarray slides 4×180K (Agilent Technologies^®^, Santa Clara, CA, USA). The microarray analysis was conducted according to manufacturer’s recommendations. Briefly, 1 μg of genomic DNA from patients and from a reference sex-matched DNA (Agilent Technologies^®^, Santa Clara, CA, USA) were fragmented with enzymatic digestion, using restriction enzymes AluI and RsaI. Patient and reference DNA were fluorescently labelled with Cy5 and Cy3, respectively. After columns purification of the labeled DNA, patients and matched reference DNA were combined and hybridized with Cot-1 DNA (1.0 mg/mL) to SurePrint G3 Human ISCA CGH+SNP 4×180K (Agilent Technologies) array slides. After hybridization and washing, microarrays slides were scanned using a SureScan Microarray Scanner (Agilent Technologies). Genomic CytoGenomics software (Agilent Technologies) was used for data extraction and quality control evaluation, and aberration reports. The ADM-2 algorithm was used to call aberrations (filtering option of a minimum 5 probes in the region, ≥100 kb size and minimum average log2 ratio > 0.3). Genomic positions were defined using GRCh38. Homozygosity regions were considered DNA sequences free of heterozygosity, with dimensions of at least 1 Mb. For the interpretation and classification of CNVs, all detected CNVs were systematically searched in the public databases, Database of Genomic Variants (http://dgvbeta.tcag.ca/dgv/app/home?ref= GRCh38 accessed on 15 October 2022), the Database of Chromosomal Imbalance and Phenotype in Humans using Ensemble Resources (DECIPHER) (https://decipher.sanger.ac.uk/ accessed on 15 October 2022), and the Database of Genomic Variants OMIM (https://www.ncbi.nlm.nih.gov/omim/ accessed on 15 October 2022), which provide cytogenetic and clinical information on large series of patients. CNVs are considered pathogenic if they are reported in publicly available databases and in the medical literature as being associated with known disease, and are likely to be clinically significant and considered as benign variants if the CNVs are recorded in publicly available genomic databases as polymorphic variants among control individuals.

Parental DNA samples were available from 3/9 patients tested with CGH-array. We also tested patient’s parents with the karyotype and MLPA test, kit P264; the results were normal.

## 3. Results

**Figure 1:**
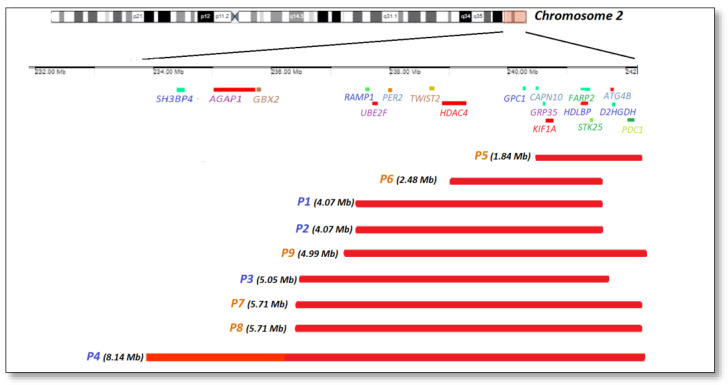
2q37 Deletion mapping that shows the size and location of the chromosomal 2q deletions found in our study. With blue, cases P1, P2, P3, and P4 are pure deletions; with orange, cases P5, P6, P7, P8, and P9 are deletions associated to other CNVs.

The MLPA tests performed initially identified the 2q37 deletion in all nine analyzed cases. Using CGH-array, we identified different deletion sizes in our cases, from 1.84 Mb to 8.14 Mb, as well as other associated CNVs (Figure 1). 

**Figure 2:**
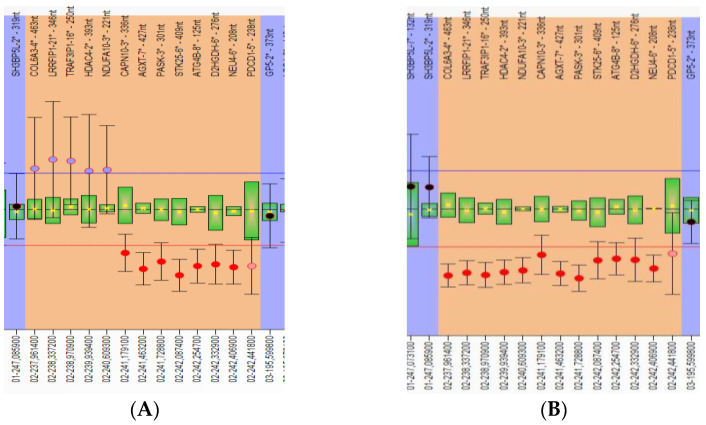
Partial images of MLPA results for follow-up probe mix P264 in bar chart format generated by Coffalyser.Net software showing the following: (**A**) Case 5—an approximately 1.7Mb (chr2:241,179,100–242,441,800-hg18) heterozygous deletion of the 2q37 subtelomeric region (ratio < 0.7), and duplication (3 copies, ratio > 1.3) in the continuation of the deleted part, possibly more extensive than the analyzed region of 3.3 Mb (chr2:237,961,400–240,609,300); (**B**) Case 9—heterozygous deletion of the 2q37 subtelomeric region (ratio < 0.7), possibly more extensive than the analyzed region of 5 Mb (chr2: 237,961,400–242,441,800-hg18).

**Figure 3:**
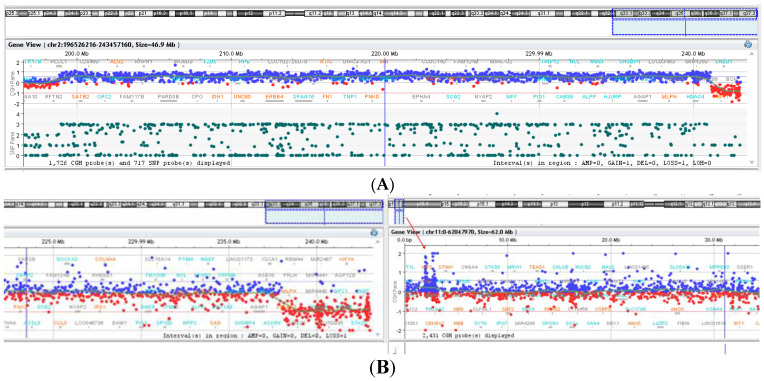
Array-CGH results generated by Agilent CytoGenomics Software: with blue, duplication; with red, deletion. (**A**) Case 5—the 1.84 Mb deletion and the approximately 42.2 Mb duplication in 2q32-q37 region arr[GRCh38] q32.1-q37.3 (198008040_240171196)x3, arr[GRCh38] 2q37.3(240256938_242098125)x1 (the enlarged view of the rearrangement). (**B**) Case 9—the 5.02 Mb deletion in 2q37 region and the 1.05 Mb subtelomeric 11p15 duplication (enlarged view): arr[GRCh38] 2q37.3(237134728_242126245)x1, arr[GRCh38] 11p15.5(1676931_2732095)x3.

**Figure 4:**
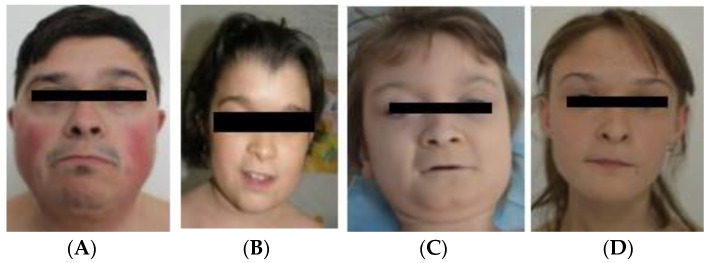
Dysmorphic face in following: (**A**) case P1 at 30 years old, (**B**) case P8 at 13 years old, (**C**) case P5 at 9 years old, (**D**) case P9 at 12 years old.

Pure 2q37 deletions have been identified in four cases: case 1 (P1) (Figure 4A), case 2 (P2), case 3 (P3), and case 4 (P4). In case P2, the chromosomal analysis identified a mosaic: 46,XX,1qh+/46,XX,1qh+,del(2q37.3)[8/30]. Deletion/duplication rearrangements were observed in the other five cases. In case P5 (Figure 4C), in addition to the 2q37.3 deletion, a 2q32.1–q37.3 duplication of 42 Mb (Figure 2A and Figure 3A) was observed that corresponded to the 2q duplication syndrome spectrum in which the most common clinical findings are similar to the 2q37 deletion: developmental delay, ID, behavioral problems, autism spectrum disorder, dysmorphic face, microcephaly, hypotonia, short stature, and underweightness [18,19,20]. 

Case P6 presented smaller 2q37.3 duplication (Figure 2B and Figure 3B) of 1.01 Mb (including 16 genes). No genes were reported as having triplosensitivity, and there are no reported cases of pathogenic phenotypes that overlap these duplications.

Cases P7 and P8 (Figure 4B) were siblings, and both associated a duplication of 6.3 Mb on 9q34.11–3.3. The duplication was reported as leading to minor anomalies, most notably developmental delay, delayed speech, hypotonia, underweightness, short stature, dysmorphic face with epicanthal folds, hypertelorism, “deep-set” eyes, short horizontal palpebral fissures, beaked nose, small mouth, low-set ears, dolichocephaly, and arachnodactyly [20,21,22].

Case P9 (Figure 4D) presented a duplication of 1.06 Mb in 11p15.5–p15.4 (Figure 2B) region that contains 37 genes).

Regarding the clinical pictures, in most of our cases we observed phenotypic aspects similar to those described for 2q37DS in the literature (Table 1). Global developmental delay and ID, of varying degrees of severity, were reported in eight out of the nine cases, and hypotonia was noted in only four cases. Only one patient presented autism spectrum disorder, whereas other behavioral problems were noticed in four cases: stereotypies, over friendly behavior, laughter crises, aggressivity, and ADHD. 

Short stature was noted in seven out of the nine cases, and obesity/overweightness only in two cases, with four other subjects being actually underweight. Craniofacial dysmorphism was suggestive for 2q37 DS in most subjects (Figure 4). 

**Figure 5:**
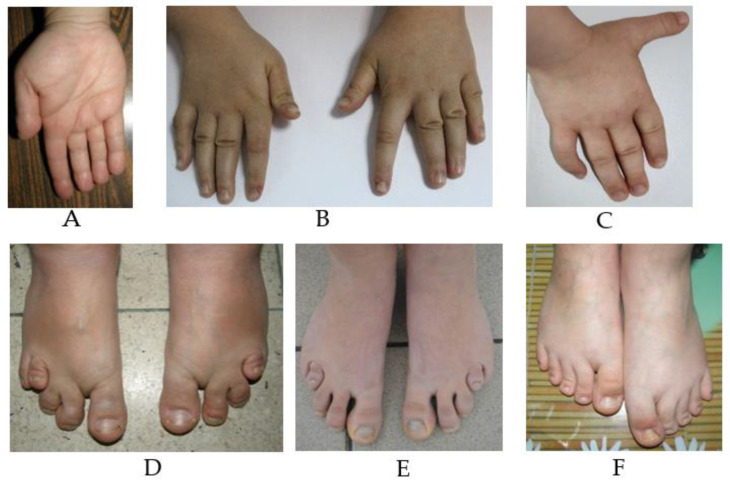
Hands and feet defects: (**A**) small puffy hands with brachydactyly type E in P1, (**B**) brachydactyly type E P2, (**C**) clinodactyly in P4, (**D**) small puffy feet, abnormal insertion of toes and brachydactyly type E in P1, (**E**) feet with brachydactyly type E—case 4, (**F**) asymmetric limbs in P8.

Skeletal anomalies were observed in eight out of the nine patients, especially malformations of the hands or feet, brachydactyly type E, asymmetric limbs or malpositioning of fingers and toes, and hypoplastic toes/fingers (Figure 5).

Heart defects were observed in four out of the nine cases, especially represented by septal defects; anomalies of the gastrointestinal system varied from mild hernia (inguinal and/or umbilical) to more severe phenotypes: intestinal malrotation, duodenal stenosis, or anorectal malformation. Moreover, in two cases, renal abnormalities were noticed; however, Wilms tumor was not identified in any case.

## 4. Discussion

Our study evaluated four new cases with pure 2q37 deletion (cases P1–P4), and five cases with deletion/duplication arrangements (cases P5–P9). In the literature, although microdeletions are better defined than microduplications, the expressions of many duplications/microduplications still remain unclear, with incomplete penetrance constantly observed. Moreover, pure duplications are quite rarely reported, which increases the difficulty of their clinical characterization. The general agreement is that the phenotypes of microduplications/partial trisomies are milder and better tolerated (in many cases no phenotypic defects are present) than deletions/microdeletions; thus, in a deletion/duplication rearrangement, the phenotype is determined mostly by the haploinsufficiency of some genes [23,24,25,26].

In our study, case P5 had a large duplication of 42 Mb on chromosome 2q32.1–q37.3, which corresponded to the 2q34 duplication syndrome. Partial 2q duplication is a very rare chromosomal abnormality usually found in chromosomal rearrangements that also involve a deletion of another partner chromosome, extremely rarely being described as pure 2q duplication (five cases reported so far). In our patient’s case, the phenotypic aspects can be attributed in part to 2q34 duplication: intellectual disability (present in all reported cases with 2q34 duplication), prominent forehead, broad nasal bridge, hypertelorism (reported in three cases in the literature with 2q duplication), thin upper lip, dental defects, clinodactyly of finger 5, and genital anomalies [27].

However, the presence of type E brachydactyly, septal defects, characteristic craniofacial dysmorphia for 2q37 deletion—aspects that are not reported in cases with 2q34 duplication—underlines the contribution of the deletion to the clinical picture.

In case P6, the associated 2q37duplication was smaller, and did not contain genes with triplosensitivity, so we can consider that the phenotype was caused only by the 2.48 Mb microdeletion in 2q37. The same situation can be applied to case P9, with a deletion of 4.99 Mb in 2q37 and a duplication of 1.06 Mb in 11 (p15.5–p15.4).

For case P7 and case P8, although they had the same rearrangement, 5.71 Mb deletion in 2q37 and 6.94 Mb duplication in 9 (q34.11–q34.3), the phenotypes were slightly different. In the literature, patients with 9q34 duplication have moderate developmental delay, limited vocabulary, hyperactivity, low birth weight, normal birth length, initial poor feeding and thriving, dolichocephaly, facial asymmetry, narrow horizontal palpebral fissures, microphthalmia, small mouth, thin upper lip with down-turned corners, musculo-skeletal defects with joint contractures, long thin limbs, and arachnodactyly. They can also present abnormal implantation of the thumb, increased space between the first and second fingers, and cardiovascular and ocular defects. Although some of these aspects were found in our cases, the phenotypes of the two siblings seemed to be more suggestive of 2q37DS [28,29].

A special case was P2, in which the chromosomal analysis identified the following mosaic: 46,XX,1qh+/46,XX,1qh+,del(2q37.3). To our knowledge, cases of mosaicism are very rare. The presence of the mosaic could be responsible for a milder phenotype; however, that was not true in our patient’s case, where the phenotypic aspects were quite characteristic for 2q37DS.

Although a syndrome with contiguous genes deletion, in 2q37DS the size of the deletion cannot be correlated with the severity of the abnormal phenotype. In some previous studies, the discordance between large deletions of 9.5–10 Mb with mild phenotype was highlighted, as well as small deletions of 828 Kb with a more complex and severe phenotype [6,30,31]. Our study underlines this aspect, not observing major phenotypic differences between small deletions of ~2 Mb (P5, P6) and large deletions of ~8 Mb (P4); the differences between the cases was given rather by the dosage sensitivity of the lost genes than by the size of the deletion. However, in the 2q37 region, not many genes have been reported as having a known dosage sensitivity.

The *HDAC4* gene has been proposed as being mainly responsible in producing the phenotypic aspects of the 2q37 microdeletion, especially brachydactyly type E, behavioral disorders, and ID—the marker clinical features in this syndrome [7,32]. The gene encodes for histone deacetylase 4, which is a class IIa HDAC that shuttles between the cytoplasm and nucleus. The nuclear localization and activity of *HDAC4* are dependent on the binding of 14-3-3 proteins. HDAC4 acts as a scaffold for other co-repressor systems, such as the N-CoR/HDAC3 complex, and represses gene expressions. Furthermore, HDAC4 represses the activity of runt-related transcription factor 2 (RUNX2) and myocyte enhancer factors (MEF2A, MEF2C). Animal studies showed that HDAC4 deficiency alone induces neurological consequences similar to 2q37 DS in humans [25,26]. Williams et al. reported two cases of intragenic *HDAC4* deletion/insertion with phenotypes similar to 2q37DS [7]. Morris et al. postulated that the severity of 2q37 DS may be due to the *HDAC4* dosage effect [31]. Moretti et al. reported three related patients with 2q37 deletions downstream of *HDAC4* who presented intellectual disability, facial dysmorphisms, microcephaly, and congenital heart defects, but no brachydactyly type E [33]. Wakeling et al. reported seven unrelated patients with heterozygous de novo missense variants *HDAC4* that impair 14-3-3 binding, and lead to a gain-of-function effect. The phenotype included significant DD/ID, seizures, distinctive facial features (hypertelorism, a full lower lip, long palpebral fissures, frontal upsweep of hair, widely spaced teeth, and large ears), scoliosis, delayed closure of the anterior fontanelle, but none of the patients had brachydactyly type E [34].

In our study, eight of the nine patients had haploinsufficiency for this gene, but only six of them had brachydactyly. Moreover, in the subject without the *HDAC4* gene loss (P5), brachydactyly type E was noted. In literature data, only 50–62% of cases had brachydactyly [32]; therefore, it was suggested that this was a variable expressivity or incomplete penetrance of the gene, as well as the existence of other genes possibly responsible for the occurrence of type E brachydactyly. In this sense, *PER2*, *TWIST2*, *GPC1* [11,12], *GPR*35 [35], *FARP2*, *STK*25, and *PDC1* [36] were proposed as candidates. In case P5, the 1.84 Mb deletion included *GPR35*, *FARP2*, *STK25*, and *PDC1* genes, which could have been potential causes of this feature in our patient.

Mild to moderate developmental delay (DD) and intellectual disability (ID) was reported in 79% of the cases with 2q37DS by Le et al. (2019), in an extensive study of 103 cases. [6]. Other previous studies reported intellectual disability and developmental delay in 100% of cases [6]. All but one of our cases presented DD and subsequently ID; however, in two cases, severe ID was noted. Some cases of 2q37 deletion with severe ID have been reported [6,30], but in two of our cases (P5, P7), the severity could also have been attributed to the associated CNVs. 

Le et al. also reported abnormal behavior in 79% of cases: autism spectrum disorder in 30% of cases, repetitive behavior in 24%, hyperactivity in 15%, aggressive behavior in 12%, delayed social skills in 10%, attention deficit disorder in 9%, and friendly disposition in 12% [6,32]. In our study, cases 5 and 9 had abnormal behavior. Contrary to other studies where a haploinsufficiency of *KIF1A* [37], *FARP2*, *HDLBP* [10], and *AGAP1* genes [13,38] was cited as a possible cause for autism in 30–35% of 2q37DS [1,3], in our patients, autistic spectrum disorder was found in just one case. This could be explained by the small number of cases in our study. Although autism was rare, other behavioral disorders (extremely friendly behavior, laughter crises, aggressiveness, and ADHD) were noted in four of the cases. 

Characteristic facial dysmorphism, noted in 86% of the cases in the literature, is also a key factor for the recognition of this syndrome. The main features found are frontal bossing (41%), broad face (35%), special arched appearance of the eyebrows (32%), shortened palpebral fissures (27% of cases), deep-set eyes (19% of subjects), a thin upper lip (39%), depressed nasal bridge (25%), prominence of the columella (18%), and hypoplastic alae (15%) [6,32]. Our findings support the statistics reported in previous studies, in that eight out of nine of our patients presented a characteristic dysmorphic face. However, we noticed in our cases some particularities or clinical features that are less often reported. Arched eyebrows were observed in seven out of nine of the patients, but five cases presented bushy eyebrows; (three other cases with bushy eyebrows were reported in other studies) [14,39]. Four cases presented a low frontal hairline, an aspect frequently described in 2q37DS (46% cases [6,38]); however, two cases presented a high frontal hairline, which has not been reported thus far in 2q37DS. Microcephaly, reported more frequently in recent studies of 2q37DS [6,39,40,41], was observed in six cases from our study. Furthermore, one case presented craniosynostosis; in the literature, another case with craniosynostosis was reported in a patient with del 2q37 associated with a duplication of 5q34 [42].

Another difference from the literature noted in our cases regarded growth parameters. While short stature was reported in the literature in 22% cases [1,32], it was noted in more than half of our cases (5/9). 2q37 DS is usually associated with overweightness or obesity (34–75% of cases), especially in deletions larger than 4.8 Mb [3,6,32]. The genes *HDAC4*, *CAPN10*, and *HDLBP* have been proposed as being responsible for the weight gain manifested since childhood (after 2 years old) [2,7], as well as the *AGAP1* and *SH3BP4* genes in larger or proximal deletions [13]. In our study, only one patient presented obesity, and one was overweight, but in four subjects, underweightness was observed (with standard deviations between −3 and −2.5 SD). Two of these underweight patients were young, and overweightness may not have set in yet. The other two had a deletion/duplication arrangement, and it is possible that their low weight was a consequence of the associated CNV or gastrointestinal malformations. As for the cases with normal weight, most of our patients were diagnosed early and kept under observation; therefore, excessive weight gain prevention was possible by maintaining a healthy diet and good management.

Heart defects are not a consistent sign in 2q37 DS, being presented in the literature in 16–20% cases [32,43]. The defect is usually mild, with mostly septal defects, less often coarctation of the aorta, or arch anomalies [2,32]. Severe cardiac anomalies are very rare in 2q37DS [1,32]. Four of our patients presented cardiac abnormalities: three had VSD, and one had a more severe cardiac malformation, with chronic heart failure, ASD, and a bicuspid aortic valve. In a study on 20 patients, Aldred et al. [1] proposed that a gene from the 3.4 Mb overlapping region between the *RAMP1* and *AC005237CA* genes is responsible for the cardiac abnormalities. Two of our cases covered this region, but the other two had a smaller deletion, suggesting a restriction of the overlapping area for cardiac defects to a region of 0.66 Mb between the *GPC1* and *STICK25* genes.

Regarding neurological aspects, hypotonia was reported by Le et al. in 27% of cases, seizures in 16%, and brain malformations in 10% (agenesis of the corpus callosum, Dandy–Walker malformation, cerebral atrophy, holoprosencephaly) [6,32]. Although in five out of nine of our cases, hypotonia in infancy was observed, and seizures in four out of nine; only one of these cases had associated brain malformations.

Other clinical aspects, described less often in previous studies but observed in our patients, were translucent skin and telangiectasias (6/9 cases). Low set nipples were also observed in seven out of nine patients (reported in literature in 13% of cases [32]). A more peculiar phenotypic feature that was found in five of our cases was a particular disposition of the adipose tissue as a hump of fat on the upper thorax. To our knowledge, this sign has not yet been described in the literature in patients with 2q37 DS.

Besides the great phenotypic variability, 2q37DS also has great molecular variability. Thus far, no common breakpoints have been established, and the minimum critical deleted region was reduced by 2–3 Mb, including several genes, but especially the *HDAC4* gene [6,7,44]. In the identification of 2q37DS, as in the rest of the syndromes with deletions and microdeletions, CGH-array is considered the gold standard. In our study, we first used MLPA as a diagnostic test, and later, for a more detailed analysis, we used CGH-array. MLPA is a feasible diagnostic test in cases with the classic phenotype, considering its low cost, and the follow-up kit allows for confirmation of deletions. In cases with a complex phenotype that would suggest the involvement of other anomalies, it is useful to perform CGH-array for the detection of other involved CNVs. Moreover, array-CGH allows determination of the size of the deletion, which is extremely useful for establishing future genotype–phenotype correlations.

## 5. Conclusions

The present study focused on finding new clinical signs that could improve the recognition of the syndrome, based on correlations between clinical molecular diagnostic traits. 

Even in the case that SNP+CGH-array technique is not available, being more expensive and complex, MLPA has the advantage to be an easy, fast, and unexpensive technique, and is a very reliable tool for the diagnosis of 2q37DS syndrome; clinicians should be confident in the diagnosis established by this technique. In this sense, we propose the use of MLPA as a screening test in all patients with a slightly suggestive phenotype for 2q37DS, as well as in their relatives, and later analysis of the deletion through a SNP+CGH-array technique to establish a more complex management. 

## Figures and Tables

**Table 1:** Clinical features in our patients.

Case	P1	P2	P3	P4	P5	P6	P7	P8	P9
Sex	m	f	f	f	m	f	m	f	f
Age (years)	30	8	4	12	9	2	18	15	12
GDD/ID	mild	moderate	-	moderate	profound	moderate	severe	moderate	moderate
Hypotonia	+	+	-	-	+	-	+	+	-
Behavioral problems	stereotipies	-	n.d.	ADHD	-	-	autism	aggressivity	laught crises
IUGR	-	-	-	-	-	+	-	-	-
Short stature	+	+	+	tall	+	+/−	+	+	+
Obesity	+	-	underweight	+	underweight	underweight	-	-	underweight
Brachydactyly E	+, MT4,5, AI	+, MT3–5	-	clinodactyly	+, tapering	AI	-	+, MC4,5	+, MC/MT4,5,
Joint hypermobility	+	+	-	-	+	-	-	-	-
Asymmetric limbs	-	-	-	-	-	-	-	+	-
Low frontal hairline	+/−	high	-	-	+	-	+, widow’s peak	+, widow’s peak	high
Frontal bossing	-	+	+	-	+	-	-	-	-
Thin/arched eyebrows	+, BE	+, BE	-	+, medial sparse	-	+, BE, medial sparse	+, BE	+, BE	+, medial sparse
Narrow PF	+	+/−	-	-	+/-	-	+	+	+
V-shaped nasal tip	-	+	-	-	-, broad	-	-	+	+
Hypoplastic alae nasi	+	+	-	+	+	+	+	+	+
Smooth philtrum	-	+	-	+/−	+	+	+	+	+
Thin upper lip	+	+	-	-	+	+	+	+	+
Full cheeks	+	-	-	-	-	+	-	-	-
Deep-set eyes	-	-	+	-	-	-	-	-	-
Microcephaly	+/−	+/−	-	-	+	++	+/−	+/−	Craniosynostosis
Short neck	+	+	+	+	+	+	+	+	+
Low-set nipples	+	+, wide-set	+	+	-	+	+	+, inverted	wide-set
Small/puffy hands/feet	+	-	+	-	-	-	+	+	+/−
TST	+	+	-	+	-	+	+	+	-
Other	gynoid aspect, kyphosis, AH, seizures, strabismus, HI, pigmented nevi, immune deficit, H	gynoid aspect, VSD, IH, UH, renal defect, AH	IH, duodenal stenosis, intestinal malrotation, LSN	gynoid aspect, AH	broad/bifid chin, ASD, BAV, hypospadias, criptorchydism, scoliosis, spastic tetraparesis	VSD, anorectal malformation, lymphedema, AH, immune deficit	kyphosis, AH, seizures, hairy knees, horshoe kidneys, H	kyphosis, asymmetric face, hairy knees seizures, vision defect, H, schizencephaly	broad/short thumb, sacral sinus, seizures
Deletion size	4.07 Mb	4.07 Mb	5.05 Mb	8.14 Mb	1.84 Mb	2.48 Mb	5.71 Mb	5.71 Mb	4.99 Mb
Cytogenetic localization	237,428,584–241,502,422	237,428,584–241,502,422	236,452,772–241,502,422	233,358,209–24,150,242	240,256,938–242,098,125	239,017,666 –241,502,423	236,452,772–24,216,033	236,452,772–24,216,033	237,134,728–242,126,245
Associated CNV	no	no(mosaic)	no	no	Dup 2 (q32.1–q37.3) 42.1 Mb	Dup 2q37.3 1.01 Mb	Dup 9(q34.11–q34.3) 6.94 Mb	Dup 9(q34.11–q34.3) 6.94 Mb	Dup 11(p15.5–p15.4) 1.06 Mb

Age at the last evaluation; ADHD = Attention-deficit/hyperactivity disorder; AI = abnormal insertion of toes; AH = upper thoracic adipose hump; ASD = atrial septal defect; BAV = bicuspid aortic valve; BE = bushy eyebrows; GDD/ID = global development delay/intellectual disability; H = hypercholesterolemia HI = hearing loss; IH = inguinal hernia; IUGR = intrauterine growth restriction; MC = metacarpal bones; MT = metatarsal bones; PF = palpebral fissures; TST = translucent skin and telangiectasias; UH = umbilical hernia; VSD = ventricular septal defect.

## Data Availability

The data presented in this study are available on request from the corresponding author. The data are not publicly available due to ethical reasons.

## References

[1] Aldred M.A., Sanford R.O.C., Thomas N.S., Barrow M.A., Wilson L.C., Brueton L.A., Bonaglia M.C., Hennekam R.C.M., Eng C., Dennis N.R. (2004). Molecular Analysis of 20 Patients with 2q37.3 Monosomy: Definition of Minimum Deletion Intervals for Key Phenotypes. J. Med. Genet..

[2] Falk R.E., Casas K.A. (2007). Chromosome 2q37 Deletion: Clinical and Molecular Aspects. Am. J. Med. Genet. C Semin. Med. Genet..

[3] Casas K.A., Mononen T.K., Mikail C.N., Hassed S.J., Li S., Mulvihill J.J., Lin H.J., Falk R.E. (2004). Chromosome 2q Terminal Deletion: Report of 6 New Patients and Review of Phenotype-Breakpoint Correlations in 66 Individuals. Am. J. Med. Genet. A.

[4] Lukusa T., Vermeesch J.R., Holvoet M., Fryns J.P., Devriendt K. (2004). Deletion 2q37.3 and Autism: Molecular Cytogenetic Mapping of the Candidate Region for Autistic Disorder. Genet. Couns. Geneva Switz..

[5] Fisch G.S., Falk R.E., Carey J.C., Imitola J., Sederberg M., Caravalho K.S., South S. (2016). Deletion 2q37 Syndrome: Cognitive-Behavioral Trajectories and Autistic Features Related to Breakpoint and Deletion Size. Am. J. Med. Genet. A.

[6] Leroy C., Landais E., Briault S., David A., Tassy O., Gruchy N., Delobel B., Grégoire M.-J., Leheup B., Taine L. (2013). The 2q37-Deletion Syndrome: An Update of the Clinical Spectrum Including Overweight, Brachydactyly and Behavioural Features in 14 New Patients. Eur. J. Hum. Genet. EJHG.

[7] Williams S.R., Aldred M.A., Der Kaloustian V.M., Halal F., Gowans G., McLeod D.R., Zondag S., Toriello H.V., Magenis R.E., Elsea S.H. (2010). Haploinsufficiency of HDAC4 Causes Brachydactyly Mental Retardation Syndrome, with Brachydactyly Type E, Developmental Delays, and Behavioral Problems. Am. J. Hum. Genet..

[8] Cho E.-K., Kim J., Yang A., Cho S.Y., Jin D.-K. (2017). 2q37 Deletion Syndrome Confirmed by High-Resolution Cytogenetic Analysis. Ann. Pediatr. Endocrinol. Metab..

[9] Doherty E.S., Lacbawan F.L., Adam M.P., Everman D.B., Mirzaa G.M., Pagon R.A., Wallace S.E., Bean L.J., Gripp K.W., Amemiya A. (1993). 2q37 Microdeletion syndrome—Retired chapter, for historical reference only. GeneReviews®.

[10] Felder B., Radlwimmer B., Benner A., Mincheva A., Tödt G., Beyer K.S., Schuster C., Bölte S., Schmötzer G., Klauck S.M. (2009). FARP2, HDLBP and PASK Are Downregulated in a Patient with Autism and 2q37.3 Deletion Syndrome. Am. J. Med. Genet. A.

[11] Smith M., Escamilla J.R., Filipek P., Bocian M.E., Modahl C., Flodman P., Spence M.A. (2001). Molecular Genetic Delineation of 2q37.3 Deletion in Autism and Osteodystrophy: Report of a Case and of New Markers for Deletion Screening by PCR. Cytogenet. Cell Genet..

[12] Syrrou M., Keymolen K., Devriendt K., Holvoet M., Thoelen R., Verhofstadt K., Fryns J.-P. (2002). Glypican 1 Gene: Good Candidate for Brachydactyly Type E. Am. J. Med. Genet..

[13] Wassink T.H., Piven J., Vieland V.J., Jenkins L., Frantz R., Bartlett C.W., Goedken R., Childress D., Spence M.A., Smith M. (2005). Evaluation of the Chromosome 2q37.3 Gene CENTG2 as an Autism Susceptibility Gene. Am. J. Med. Genet. Part B Neuropsychiatr. Genet. Off. Publ. Int. Soc. Psychiatr. Genet..

[14] Vlad C.-E., Foia L.G., Popescu R., Popa I., Aanicai R., Reurean-Pintilei D., Toma V., Florea L., Kanbay M., Covic A. (2021). Molecular Genetic Approach and Evaluation of Cardiovascular Events in Patients with Clinical Familial Hypercholesterolemia Phenotype from Romania. J. Clin. Med..

[15] Gavril E.-C., Popescu R., Nucă I., Ciobanu C.-G., Butnariu L.I., Rusu C., Pânzaru M.-C. (2022). Different Types of Deletions Created by Low-Copy Repeats Sequences Location in 22q11.2 Deletion Syndrome: Genotype-Phenotype Correlation. Genes.

[16] Gavril E.-C., Luca A.C., Curpan A.-S., Popescu R., Resmerita I., Panzaru M.C., Butnariu L.I., Gorduza E.V., Gramescu M., Rusu C. (2021). Wolf-Hirschhorn Syndrome: Clinical and Genetic Study of 7 New Cases, and Mini Review. Child. Basel Switz..

[17] Butnariu L., Rusu C., Caba L., Pânzaru M., Braha E., Grămescu M., Popescu R., Bujoranu C., Gorduza E.V. (2013). Genotype- Phenotype Correlation in Trisomy X: A Retrospective Study of a Selected Group of 36 Patients and Review of Literature. Rev. Med. Chir. Soc. Med. Nat. Iasi.

[18] Bird L.M., Mascarello J.T. (2001). Chromosome 2q Duplications: Case Report of a de Novo Interstitial Duplication and Review of the Literature. Am. J. Med. Genet..

[19] Usui D., Shimada S., Shimojima K., Sugawara M., Kawasaki H., Shigematu H., Takahashi Y., Inoue Y., Imai K., Yamamoto T. (2013). Interstitial Duplication of 2q32.1-Q33.3 in a Patient with Epilepsy, Developmental Delay, and Autistic Behavior. Am. J. Med. Genet. A.

[20] Abolhasani M., Vasei M., Safavi M. (2019). A Rare Case of Duplication of Chromosome 2 (Q31.3q36.3) in a 4.5-Year-Old Boy and Review of the Literature. Int. J. Pediatr..

[21] Moody A., Athikarisamy S.E., Yeung A., Burgess T., Malhotra A. (2013). Neonatal Presentation of Chromosome 9q33.2-Q34.3 Duplication. Gene.

[22] Liu J., Hu H., Ma N., Jia Z., Zhou Y., Hu J., Wang H. (2016). A de Novo Duplication of Chromosome 9q34.13-Qter in a Fetus with Tetralogy of Fallot Syndrome. Mol. Cytogenet..

[23] Watson C.T., Marques-Bonet T., Sharp A.J., Mefford H.C. (2014). The Genetics of Microdeletion and Microduplication Syndromes: An Update. Annu. Rev. Genomics Hum. Genet..

[24] Vissers L.E.L.M., Stankiewicz P. (2012). Microdeletion and Microduplication Syndromes. Methods Mol. Biol. Clifton NJ.

[25] Wou K., Levy B., Wapner R.J. (2016). Chromosomal Microarrays for the Prenatal Detection of Microdeletions and Microduplications. Clin. Lab. Med..

[26] Liehr T., Ewers E., Hamid A.B., Kosyakova N., Voigt M., Weise A., Manvelyan M. (2011). Small Supernumerary Marker Chromosomes and Uniparental Disomy Have a Story to Tell. J. Histochem. Cytochem..

[27] Suzuki T., Osaka H., Miyake N., Fujita A., Uchiyama Y., Seyama R., Koshimizu E., Miyatake S., Mizuguchi T., Takeda S. (2022). Distal 2q Duplication in a Patient with Intellectual Disability. Hum. Genome Var..

[28] Spinner N.B., Lucas J.N., Poggensee M., Jacquette M., Schneider A. (1993). Duplication 9q34→qter Identified by Chromosome Painting. Am. J. Med. Genet..

[29] Bonati M.T., Castronovo C., Sironi A., Zimbalatti D., Bestetti I., Crippa M., Novelli A., Loddo S., Dentici M.L., Taylor J. (2019). 9q34.3 Microduplications Lead to Neurodevelopmental Disorders through EHMT1 Overexpression. Neurogenetics.

[30] Safi S., Yamasaki T., Glidden D., Sanders S., Carreon C. (2022). 2q37.3 Deletion with Complex Heart Defects Suggesting Interruption of Early Ventricular Looping. Congenit. Heart Dis..

[31] Morris B., Etoubleau C., Bourthoumieu S., Reynaud-Perrine S., Laroche C., Lebbar A., Yardin C., Elsea S.H. (2012). Dose Dependent Expression of HDAC4 Causes Variable Expressivity in a Novel Inherited Case of Brachydactyly Mental Retardation Syndrome. Am. J. Med. Genet. A.

[32] Le T.N., Williams S.R., Alaimo J.T., Elsea S.H. (2019). Genotype and Phenotype Correlation in 103 Individuals with 2q37 Deletion Syndrome Reveals Incomplete Penetrance and Supports HDAC4 as the Primary Genetic Contributor. Am. J. Med. Genet. A.

[33] Moretti P.N., Figueiredo A.C.V., Saliba A., Versiani B.R., Oliveira S.F., Pic-Taylor A., Mazzeu J.F. (2020). Genotype and Phenotype Correlation in a Family with a 2q37 Deletion Downstream of *HDAC4*. Am. J. Med. Genet. A.

[34] Wakeling E., McEntagart M., Bruccoleri M., Shaw-Smith C., Stals K.L., Wakeling M., Barnicoat A., Beesley C., Hanson-Kahn A.K., Kukolich M. (2021). Missense Substitutions at a Conserved 14-3-3 Binding Site in HDAC4 Cause a Novel Intellectual Disability Syndrome. Hum. Genet. Genom. Adv..

[35] Shrimpton A.E., Braddock B.R., Thomson L.L., Stein C.K., Hoo J.J. (2004). Molecular Delineation of Deletions on 2q37.3 in Three Cases with an Albright Hereditary Osteodystrophy-like Phenotype. Clin. Genet..

[36] Davids M.S., Crawford E., Weremowicz S., Morton C.C., Copeland N.G., Gilbert D.J., Jenkins N.A., Phelan M.C., Comb M.J., Melnick M.B. (2001). STK25 Is a Candidate Gene for Pseudopseudohypoparathyroidism. Genomics.

[37] Devillard F., Guinchat V., Moreno-De-Luca D., Tabet A.-C., Gruchy N., Guillem P., Nguyen Morel M.-A., Leporrier N., Leboyer M., Jouk P.-S. (2010). Paracentric Inversion of Chromosome 2 Associated with Cryptic Duplication of 2q14 and Deletion of 2q37 in a Patient with Autism. Am. J. Med. Genet. A.

[38] Pacault M., Nizon M., Pichon O., Vincent M., Le Caignec C., Isidor B. (2019). A de Novo 2q37.2 Deletion Encompassing AGAP1 and SH3BP4 in a Patient with Autism and Intellectual Disability. Eur. J. Med. Genet..

[39] Kariminejad A., Kariminejad R., Tzschach A., Ullmann R., Ahmed A., Asghari-Roodsari A., Salehpour S., Afroozan F., Ropers H.-H., Kariminejad M.H. (2009). Craniosynostosis in a Patient with 2q37.3 Deletion 5q34 Duplication: Association of Extra Copy of MSX2 with Craniosynostosis. Am. J. Med. Genet. A.

[40] Gürsoy S., Kutbay Y.B., Özdemir T.R., Hazan F. (2019). The Clinical and Molecular Features of Three Turkish Patients with a Rare Genetic Disorder: 2q37 Deletion Syndrome. Turk. J. Pediatr..

[41] Arefzadeh A., Khalighinejad P., Ataeinia B., Parvar P. (2018). Brachydactyly Mental Retardation Syndrome with Growth Hormone Deficiency. Endocrinol. Diabetes Metab. Case Rep..

[42] Zaki A., Shaheen N., Ramadan A., Ramadan A., Nashwan A. (2022). A Rare Case of 2q37 Deletion Syndrome Presented with Patent Foramen Ovale. Clin. Case Rep..

[43] Mehraein Y., Pfob M., Steinlein O., Aichinger E., Eggert M., Bubendorff V., Mannhart A., Müller S. (2015). 2q37.3 Deletion Syndrome: Two Cases with Highly Distinctive Facial Phenotype, Discordant Association with Schizophrenic Psychosis, and Shared Deletion Breakpoint Region on 2q37.3. Cytogenet. Genome Res..

[44] Fallah M.S., Szarics D., Robson C.M., Eubanks J.H. (2021). Impaired Regulation of Histone Methylation and Acetylation Underlies Specific Neurodevelopmental Disorders. Front. Genet..

